# Clinical outcomes of patients with unilateral internal derangement of the temporomandibular joint following arthrocentesis and stabilization splint therapy

**DOI:** 10.1186/s40902-024-00436-7

**Published:** 2024-07-08

**Authors:** Hyun-A Heo, Suhyun Park, Sung-Woon Pyo, Hyun-Joong Yoon

**Affiliations:** 1 Department of Advanced General Dentistry, Bucheon St. Mary’s Hospital, College of Medicine, The Catholic University of Korea, Seoul, Republic of Korea; 2 Department of Oral and Maxillofacial Surgery, Bucheon St. Mary’s Hospital, College of Medicine, The Catholic University of Korea, Seoul, Republic of Korea

**Keywords:** Arthrocentesis, Stabilization splint, Internal derangement, Temporomandibular joint disorder

## Abstract

**Background:**

The management of internal derangement (ID) of the TMJ is challenging because of multiple etiologic factors and varying degrees of severity. The aim of this study was to evaluate the clinical outcomes of patients with unilateral ID treated with arthrocentesis and stabilization splint therapy during a 6-month period.

**Methods:**

A total of 105 patients (87 females, 18 males) with unilateral ID were included in this study. Patients were divided into unilateral anterior disc displacement with reduction (ADDwR) and unilateral anterior disc displacement without reduction (ADDwoR). Patients with ADDwoR were subdivided according to the erosive bone changes. Objective parameters on mandibular movement and subjective parameters on pain were obtained and assessed.

Their clinical outcomes before and after arthrocentesis and stabilization splint therapy were compared with the chi-square, Fisher’s exact test, paired *t*-test, or Wilcoxon singed-rank test.

**Results:**

All objective parameters of unilateral ID patients significantly increased at the 6-month follow-up. The differences in mean visual analog scale (VAS) pain scores were statistically significant in all subjective variables (*p* < 0.01). In joints with ADDwoR, preoperative maximal mouth opening, and maximal protrusive movement in both groups, with erosive and non-erosive changes were significantly increased after 6 months (*p* < 0.01). However, right and left maximal lateral movement increased after treatment in both groups but without significant differences. All VAS pain scores on jaw movement and palpation of associated muscles showed a significant decrease regardless of erosive changes.

**Conclusions:**

The combination of arthrocentesis and subsequent stabilization splint therapy was shown to be highly effective in pain reduction and improvement of mandibular movements in both unilateral ADDwR and ADDwoR, as well as in cases with both erosive and non-erosive bony changes associated with unilateral ADDwoR.

## Background

Internal derangement (ID) of the temporomandibular joint (TMJ) is the most frequent TMJ disorder and is defined as disc displacement from its normal position during joint function [1–3]. The ID of the TMJ can be classified into two main groups based on disc displacement with and without reduction. According to the updated 2014 Diagnostic Criteria for TMDs (DC/TMDs), it can be divided into four stages: disc displacement with reduction (DDwR), DDwR with intermittent locking, disc displacement without reduction (DDwoR) with limited opening, and DDwoR without limited opening [4].

Clinical signs and symptoms of ID generally include joint sounds (clicking, popping), limitation of mouth opening, deflexion or deviation of the mandible during mouth opening, and pain involving the TMJ and masticatory muscles. Advanced stages of DDwoR are characterized by crepitation and degenerative changes such as disc perforation, condylar resorption, and osteoarthritis [5, 6].

Magnetic resonance imaging (MRI) is considered the standard imaging technique for soft tissue structures, such as the articular disc of the TMJ region. Yang et al. [7] reported that MRI can be successfully used to distinguish disc displacement with or without reduction as well as to assess anterior disc displacement.

Treatment methods for ID basically consist of conservative and surgical methods [8]. Conservative treatments include occlusal splints, physiotherapy, and medication, and are the most frequently used in the early stages of ID [9]. Surgical treatments such as arthrocentesis and arthroscopy can be considered for cases refractory to conservative management or in cases of severe internal derangement. In ID patients showing no improvement with previous minimally invasive treatments or having significant degenerative changes, open joint surgery may be considered a viable treatment option [6].

Ever since the introduction of arthrocentesis by Nitzan et al. [10], it has become a widespread surgical therapy for ID due to its minimally invasive characteristic and high success rates in various cases [11, 12]. However, arthrocentesis alone is not enough because the lack of control of mechanical stress on the TMJ usually leads to treatment failure.

Tatli et al. [5] emphasized that conservative and surgical options should be considered concurrently. Our previous study [13] also demonstrated successful outcomes with arthrocentesis and subsequent stabilization splint therapy in patients with anterior disc displacement without reduction (ADDwoR).

The aim of our study was to evaluate the clinical outcomes of patients with unilateral internal derangements, including anterior disc displacement (ADD) with and without reduction following consecutive arthrocentesis and stabilization splint therapy. The second aim was to compare the effectiveness of this treatment protocol in patients with unilateral ADD without reduction, with or without bony changes.

## Materials and methods

This study was approved and informed consent was waived by the Institutional Review Board of the Catholic University of Korea (Approval number: HC20RIDI0104). One hundred five patients (87 females, 18 males), who were followed by the Department of Oral and Maxillofacial Surgery, at Yeouido and Bucheon St. Mary’s Hospital, the Catholic University of Korea, Republic of Korea, from November 2005 to August 2020, were enrolled in this retrospective study.

The patients were selected based on a retrospective review of their records and MRI results. We included only cases of unilateral ID. Patients were divided into two groups: unilateral anterior disc displacement with reduction (ADDwR) and anterior disc displacement without reduction (ADDwoR). Patients in the ADDwoR category were further subdivided according to erosive bone changes.

Patients with any systemic connective tissue diseases, a history of major jaw trauma, and a history of TMJ treatment were excluded from this study.

Objective and subjective parameters were recorded pre-operatively and at 6 months postoperatively by the same clinician. The objective parameters were maximal mouth opening (MMO), right maximal lateral movement (RLM), left maximal lateral movement (LLM), and maximal protrusive movement (PM). Subjective parameters were visual analog scale (VAS; 0–100) pain score during MMO, RLM, LLM, and PM and VAS pain score during palpation of temporalis, masseter, sternocleidomastoid (SCM), and trapezius muscles at rest. MMO was measured between the edges of the upper and lower central incisors with a millimeter ruler. Horizontal distance between midpoints of upper and lower incisors during RLM, LLM, and PM were measured in the same way. The palpation examination was carried out at reference points for given muscles, including temporal muscles (muscular 3 points: anterior, medial, and posterior bellies), masseter muscles (muscular 3 points: inferior part of the superficial belly, anterior part of the superficial belly, and deep belly), sternocleidomastoid muscles, and trapezius muscles. These muscles were examined symmetrically. All suspected muscles were examined using the tip of the index finger for at least 10 to 20 s. The fingernail should blanch. Some muscles, such as the masseter, sternocleidomastoid, and upper trapezius muscles, can be palpated between the index finger and thumb with pincer-type palpation.

MRI (Achieva, Philips Medical, Best, The Netherlands; slice thickness 2.3 mm) was used to assess internal derangement, joint effusion, and bony changes. Effusion in the MRIs was classified into four grades according to the amount of joint fluid using the grading system of Larheim et al. [14]: absent or minimal fluid = 1; moderate fluid = 2; marked fluid = 3; and extensive fluid = 4.

### Arthrocentesis and stabilization splint therapy

Arthrocentesis was performed in all patients under conscious sedation combined with local anesthesia by the same surgeon. The two-needle technique was used for lavage in the superior joint space and lactated Ringer’s solution or saline was used as the irrigation fluid. During the procedure, the jaw was manipulated repeatedly to reach the maximal opening. At the end of the procedure, 1.2 mL of hyaluronic acid (Guardix-sol; Hanmi Medicare, Seoul, Republic of Korea) was injected into the joint cavity as the therapeutic drug. Following arthrocentesis, each patient was instructed to wear a hard acrylic stabilization splint on the maxilla for 8–10 h daily for 6 months. Regular follow-up was conducted every other week to adjust the splint and to assess improvement in signs and symptoms until the study was completed.

### Statistical analysis

All statistical analyses were performed using SAS Version 9.4 (SAS Institute, Cary, NC, USA). A P-value less than 0.05 was considered statistically significant. The normal distributions of the data were assessed using the Shapiro–Wilk test. For baseline characteristics analysis of patients, the chi-square or Fisher’s exact test was used for categorical variables, and *t*-test or Wilcoxon rank sum test was used for continuous variables. To evaluate the statistical significance of treatment outcomes, either the paired *t*-test or Wilcoxon signed rank test was conducted. The data are presented as mean ± SD, median (range), and *n* (%).

## Results

A total of 105 patients (mean age, 38 years; age range, 12–81 years) received unilateral arthrocentesis and subsequent stabilization splint therapy. Females constituted 87% of the patients (*n* = 87) and males 18% (*n* = 18). Of the 105 patients, 82 were diagnosed with ADDwoR, and 23 were diagnosed with ADDwR. Baseline characteristics of the patients such as clenching, bruxism, type of occlusion, and distribution of joint effusion (JE) are summarized in Table 1. Despite the absence of a significant association between the type of disc displacement and joint effusion, 66% of all patients with ADD showed extensive effusion (Grade 4), which was higher in patients with unilateral ADDwoR than in those with unilateral ADDwR.

**Table 1:** Baseline characteristics of patients with unilateral anterior disc displacement (ADD) (*n* = 105)

Characteristics	Total (*n* = 105)	ADD without reduction (*n* = 82)	ADD with reduction (*n* = 23)	*P* value
Age, years, mean ± SD	38 ± 19.3	38.7 ± 19.6	35.2 ± 18.1	0.609
Sex, *n* (%)				0.537
Female	87 (82.9)	69 (84.2)	18 (78.3)	
Male	18 (17.1)	13 (15.9)	5 (21.7)	
Clenching, *n* (%)	30 (28.6)	23 (28.1)	7 (30.4)	0.823
Bruxism, *n* (%)	15 (14.3)	10 (12.2)	5 (21.7)	0.311
Occlusion, *n* (%)				0.051
Class I	80 (76.2)	61 (74.4)	19 (82.6)	
Class II	11 (10.5)	7 (8.5)	4 (17.4)	
Class III	14 (13.3)	14 (17.1)	–	
Joint effusion, *n* (%)				0.061
1 (no or minimal fluid)	6 (5.7)	4 (4.9)	2 (8.7)	
2 (moderate fluid)	13 (12.4)	7 (8.5)	6 (26.1)	
3 (marked fluid)	20 (19.1)	15 (18.3)	5 (21.7)	
4 (extensive fluid)	66 (62.9)	56 (68.3)	10 (43.5)	

Data are presented as mean ± SD, *n* (%)

*ADD* anterior disc displacement

*P* values were calculated using chi-square or Fisher’s exact test for categorical variables and t-test or Wilcoxon rank sum test for continuous variables

Table 2 shows the changes in objective and subjective parameters of patients with unilateral ADD after treatment. All objective parameters, including MMO, PM, RLM, and LLM were significantly increased. The differences in mean VAS pain scores before arthrocentesis and 6 months later were statistically significant in all subjective variables (*p* < 0.01) (Table 2).

**Table 2:** Changes in MMO, PM, RLM, LLM, and VAS pain scores of patients with unilateral anterior disc displacement (*n* = 105)

	Baseline	Sixth month	*P* value
MMO (mm)			** < 0.001**
Mean ± SD	40.3 ± 9.1	47.1 ± 5.4	
Median (min, max)	40 (21, 60)	47 (35, 60)	
PM (mm)			** < 0.001**
Mean ± SD	6.5 ± 2.5	7.5 ± 0.3	
Median (min, max)	7 (0, 12)	8 (2, 13)	
RLM (mm)			**0.008**
Mean ± SD	8 ± 2.5	8.6 ± 2.2	
Median (min, max)	8 (0, 15)	8 (3, 13)	
LLM (mm)			**0.009**
Mean ± SD	8.2 ± 2.8	9 ± 2.4	
Median (min, max)	8 (2, 15)	9 (2, 20)	
Pain in TMJ during MMO	35.5 ± 28.2	3.7 ± 8.8	** < 0.001**
Pain in TMJ during PM	18.1 ± 23.6	1.1 ± 4.7	** < 0.001**
Pain in TMJ during RLM	15.1 ± 23.6	1.6 ± 5.2	** < 0.001**
Pain in TMJ during LLM	12.7 ± 20.7	1.5 ± 5.5	** < 0.001**
Pain on temporalis	16.8 ± 26.4	3.6 ± 13.4	** < 0.001**
Pain on SCM	11 ± 23.7	2.8 ± 12.4	** < 0.001**
Pain on trapezius	12.6 ± 25.7	4 ± 15.1	** < 0.001**
Pain on masseter	16.1 ± 26.6	3.6 ± 10.8	** < 0.001**

Data are presented as mean ± SD, median (range)

*MMO* maximal mouth opening, *PM* maximal protrusive movement, *RLM* right maximal lateral movement, *LLM* left maximal lateral movement, *VAS* visual analog scale, *TMJ* temporomandibular joint, *SCM* sternocleidomastoid

Values in bold indicate statistically significant differences with a *P* value < .05

*P* values were calculated using paired t-test or Wilcoxon signed rank test

Table 3 summarizes the baseline characteristics of patients with unilateral ADDwoR divided into two groups based on erosive bone changes involving the TMJ. Of the 82 ADDwoR cases, 43 showed bone changes in TMJ, and 39 did not. The results showed that age was a significant factor in degenerative changes (*p* < 0.01). Extensive accumulation of joint fluid was observed in both groups.

**Table 3:** Baseline characteristics of patients with unilateral anterior disc displacement without reduction (*n* = 82)

Characteristics	No erosive change (*n* = 39)	Erosive change (*n* = 43)	*P* value
Age, years, mean ± SD	32.1 ± 19.1	44.7 ± 18.3	**0.003**
Sex, *n* (%)			0.621
Female	32 (82.1)	37 (86.1)	
Male	7 (18)	6 (14)	
Clenching, *n* (%)	7 (18)	16 (37.2)	0.053
Bruxism, *n* (%)	4 (10.3)	6 (14)	0.741
Occlusion, *n* (%)			0.750
Class I	28 (71.8)	33 (76.7)	
Class II	3 (7.7)	4 (9.3)	
Class III	8 (20.5)	6 (14)	
Joint effusion, *n* (%)			0.056
1 (no or minimal fluid)	–	4 (9.3)	
2 (moderate fluid)	1 (2.6)	6 (14)	
3 (marked fluid)	8 (20.5)	7 (16.3)	
4 (extensive fluid)	30 (76.9)	26 (60.5)	

Data are presented as mean ± SD, *n* (%)

Values in bold indicate statistically significant differences with a *P* value < .05

*P* values were calculated using chi-square or Fisher’s exact test for categorical variables and *t*-test or Wilcoxon rank sum test for continuous variables

Preoperative MMO and PM in both erosive and non-erosive cases were significantly increased after 6 months (*p* < 0.01). However, RLM and LLM were increased after treatment but did not show any significant differences in either group.

All VAS pain scores involving jaw movement and palpation of associated muscles showed significant decreases regardless of the erosive changes (Table 4).

**Table 4:** Changes in MMO, PM, RLM, LLM, and VAS pain scores of patients with unilateral anterior disc displacement without reduction

	No erosive change (baseline)	No erosive change (6 months)	*P* value	Erosive change (baseline)	Erosive change (6 months)	*P* value
MMO (mm)			** < 0.001**			** < 0.001**
Mean ± SD	38 ± 8.6	46.8 ± 5		39.7 ± 9.2	46.6 ± 5.2	
Median (min, max)	38 (23, 55)	46 (39, 60)		40 (21, 60)	47 (37, 58)	
PM (mm)			**0.003**			**0.003**
Mean ± SD	6.2 ± 2.2	7.3 ± 1.9		6.4 ± 2.8	7.5 ± 2.6	
Median (min, max)	6 (2, 11)	7 (2, 12)		7 (0, 12)	8 (2, 12)	
RLM (mm)			0.129			0.193
Mean ± SD	7.8 ± 2.4	8.5 ± 2.1		8.3 ± 2.6	8.6 ± 2.1	
Median (min, max)	8 (3, 13)	9 (4, 13)		8 (2, 15)	9 (3, 13)	
LLM (mm)			0.11			**0.03**
Mean ± SD	8.1 ± 2.4	8.9 ± 2.7		8 ± 2.9	8.9 ± 2.3	
Median (min, max)	8 (4, 15)	9 (5, 20)		8 (2, 14)	10 (2, 14)	
Pain in TMJ during MMO	39.5 ± 26.8	5.2 ± 1.1	** < 0.001**	40.5 ± 27.4	2.9 ± 6.9	** < 0.001**
Pain in TMJ during PM	20.1 ± 3	1.7 ± 7.1	** < 0.001**	21.9 ± 26.7	0.8 ± 2.7	** < 0.001**
Pain in TMJ during RLM	18.7 ± 22.8	1.4 ± 4.7	** < 0.001**	13.1 ± 25.4	2.3 ± 6.5	**0.009**
Pain in TMJ during LLM	13.2 ± 21.1	0.1 ± .8	** < 0.001**	14.4 ± 21.1	3.1 ± 8.2	** < 0.001**
Pain on temporalis	17.7 ± 28.1	4.6 ± 13.5	**0.002**	14.2 ± 22.6	1.4 ± 9.1	**0.001**
Pain on SCM	10.8 ± 23.7	4.9 ± 16.5	**0.047**	9.8 ± 22.9	0 ± 0	**0.008**
Pain on trapezius	14.6 ± 28.9	4.6 ± 15.4	**0.004**	10.9 ± 23.5	2.6 ± 11.8	**0.004**
Pain on masseter	17.9 ± 27.1	4.7 ± 15.1	**0.005**	15.3 ± 26.4	2.3 ± 7.2	**0.001**

Data are presented as mean ± SD, *n* (%)

*MMO* maximal mouth opening, *PM* maximal protrusive movement, *RLM* right maximal lateral movement, *LLM* left maximal lateral movement, *VAS* visual analog scale, *TMJ* temporomandibular joint, *SCM* sternocleidomastoid

Values in bold indicate statistically significant differences with a *P* value < .05

*P* values were calculated using paired *t*-test or Wilcoxon signed rank test

## Discussion

Although data indicate that many people with various TMDs will improve over time, even without intervention, the management of ID is considered challenging due to multiple etiological factors and the varying severity of damage [15], ranging from painless disc displacement with reduction to advanced disc displacement with severe degenerative changes.

According to the guidelines issued by the American Society of Temporomandibular Joint Surgeons in 2001, the aims of ID treatment should be (1) reduction of pain, (2) improvement of dysfunction, and (3) slowing the progression of internal derangement/osteoarthritis [16].

Arthrocentesis has been reported to be effective, minimally invasive, and safe in managing the symptoms of ID of TMJ [17, 18]. Tozoglu et al. summarized the important effects of arthrocentesis in their review article, including the elimination of inflamed synovial fluid, release of the disc, pain reduction, and joint mobilization through washing the upper joint space [17]. Nitzan also cited numerous studies reporting reduced TMJ pain and improved function after arthrocentesis [19]. Some studies have shown that arthrocentesis is significantly more effective than conservative treatment for both pain reduction and improvement of MMO, and they suggested the use of arthrocentesis as an efficient first-line treatment [20–22]. In Tang et al.’s study with over 5 years of long-term follow-up, arthrocentesis was also found to provide greater pain relief and functional improvement compared to nonsurgical interventions as initial treatment for TMJ arthralgia [25].

Stabilization splints have been reported to show therapeutic effects of balancing the occlusion, relaxation of muscle spasms, and facilitating alignment of the dislocated disc [6]. For managing ID, joint unloading is also necessary, especially in the presence of bruxism and clenching. In such cases, stabilization splints can be successfully used for joint unloading by changing the intra-articular distance and assisting patients in recognizing their bad habits [23].

Tatli et al. reported that stabilization splint therapy alone had a 60% success rate, while both arthrocentesis alone and combination therapy had success rates of over 90% in patients with disc displacement without reduction [5].

In our study, 14% of patients showed bruxism and 29% manifested clenching.

Therefore, we selected the combination treatment protocol and evaluated the treatment outcomes after arthrocentesis and stabilization splint successively in patients at various stages of unilateral TMJ ID. We diagnosed bruxism and/or clenching based on patient reports and clinical interviews. However, diagnosing via questionnaire alone may be inaccurate, as up to 80% of patients may be unaware of bruxism.

All patients underwent a single session of arthrocentesis, and none of the patients experienced complications due to the arthrocentesis procedure.

The treatment protocol resulted in improved jaw movement and pain relief. Especially, noteworthy is the dramatic reduction in pain, one of the main reasons for TMD treatment, in all patients with unilateral ADD after 6 months of treatment (*p* < 0.01). We also compared the treatment outcomes of patients diagnosed with unilateral ADDwoR with and without erosive bony changes. As a result, both groups showed significant improvement in pain, MMO, and PM, without any differences between groups. The results of the present study are consistent with those reported in the literature [9, 13, 24].

Previously reported studies [9, 13, 24] of combination therapy showed limited indications, ADDwoR. However, we applied the treatment protocol to various stages of TMJ disc displacement and conducted arthrocentesis first for quicker improvement of patients’ discomforts, such as pain and mouth opening limitation.

The strength of the present study is uniformity of patient selection and follow-up time. We included only unilateral ID with a 6-month follow-up to facilitate accurate assessment. However, this is a retrospective study and only short-term results were analyzed. The majority of patients showed ADD without reduction (78%). Thus, well-designed prospective RCTs with long-term follow-up are needed.

## Conclusions

In summary, arthrocentesis and subsequent stabilization splint therapy are highly effective in pain reduction and improvement of mandibular movements in patients with both unilateral ADDwR and ADDwoR, as well as in cases with both erosive and non-erosive bony changes associated with unilateral ADDwoR. Therefore, we suggest the combination protocol as a reliable treatment modality for ID of the TMJ.

## Data Availability

The datasets used and/or analyzed during the current study are available from the corresponding author on reasonable request.

## Abbreviations

ADDwR: Anterior disc displacement with reduction
ADDwoR: Anterior disc displacement without reduction
DDwr: Disc displacement with reduction
DDwoR: Disc displacement without reduction
ID: Internal derangement
JE: Joint effusion
LLM: Left maximal lateral movement
MMO: Maximal mouth opening
MRI: Magnetic resonance imaging
PM: Maximal protrusive movement
RLM: Right maximal lateral movement
SCM: Sternocleidomastoid
TMJ: Temporomandibular joint
VAS: Visual analog scale

## References

[1] Zhuo Z, Cai XY (2016). Radiological follow-up results of untreated anterior disc displacement without reduction in adults. Int J Oral Maxillofac Surg.

[2] Efeoglu C, Calis AS, Koca H, Yuksel E (2018). A stepped approach for the management of symptomatic internal derangement of the temporomandibular joint. J Otolaryngol Head Neck Surg.

[3] Manfredini D, Guarda-Nardini L, Winocur E, Piccotti F, Ahlberg J, Lobbezoo F (2011). Research diagnostic criteria for temporomandibular disorders: a systematic review of axis I epidemiologic findings. Oral Surg Oral Med Oral Pathol Oral Radiol Endod.

[4] Schiffman E, Ohrbach R, Truelove E (2014). Diagnostic criteria for temporomandibular disorders (DC/TMD) for clinical and research applications: recommendations of the international RDC/TMD consortium network and orofacial pain special interest group. J Oral Facial Pain Headache.

[5] Tatli U, Benlidayi ME, Ekren O, Salimov F (2017). Comparison of the effectiveness of three different treatment methods for temporomandibular joint disc displacement without reduction. Int J Oral Maxillofac Surg.

[6] Tatli U, Machon V. Internal derangements of the temporomandibular joint: Diagnosis and Management. [Internet] I Temporomandibular Joint Pathology - Current Approaches and Understanding. InTech; 2018. Available from: 10.5772/intechopen.72585.

[7] Yang Z, Wang M, Ma Y (2017). Magnetic resonance imaging (MRI) evaluation for anterior disc displacement of the temporomandibular joint. Med Sci Monit.

[8] Tvrdy P, Heinz P, Pink R (2015). Arthrocentesis of the temporomandibular joint: a review. Biomed Pap Med Fac Univ Palacky Olomouc Czech Repub.

[9] Tvrdy P, Heinz P, Zapletalova J, Pink R, Michl P (2015). Effect of combination therapy of arthrocentesis and occlusal splint on nonreducing temporomandibular joint disk displacement. Biomed Pap Med Fac Univ Palacky Olomouc Czech Repub.

[10] Nitzan DW, Dolwick MF, Martinez GA (1991). Temporomandibular joint arthrocentesis: a simplified treatment for severe, limited mouth opening. J Oral Maxillofac Surg.

[11] Kuruvilla VE, Prasad K (2012). Arthrocentesis in TMJ internal derangement: a prospective study. J Maxillofac Oral Surg.

[12] Chandrashekhar VK, Kenchappa U, Chinnannavar SN, Singh S (2015). Arthrocentesis a minimally invasive method for TMJ disc disorders - a prospective study. J Clin Diagn Res.

[13] Heo HA, Yoon HJ (2020). Clinical outcomes of patients with bilateral anterior disc displacement without reduction and erosive change of the temporomandibular joint after performance of unilateral arthrocentesis and stabilisation splint therapy. J Oral Rehabil.

[14] Larheim TA, Westesson PL, Sano T (2001). MR grading of temporomandibular joint fluid: association with disk displacement categories, condyle marrow abnormalities and pain. Int J Oral Maxillofac Surg.

[15] Wilkes CH (1989). Internal derangements of the temporomandibular joint. Pathological variations. Arch Otolaryngol Head Neck Surg.

[16] American Society of Temporomandibular Joint Surgeons (2003). Guidelines for diagnosis and management of disorders involving the temporomandibular joint and related musculoskeletal structures. Cranio.

[17] Tozoglu S, Al-Belasy FA, Dolwick MF (2011). A review of techniques of lysis and lavage of the TMJ. Br J Oral Maxillofac Surg.

[18] Nitzan DW, Price A (2001). The use of arthrocentesis for the treatment of osteoarthritic temporomandibular joints. J Oral Maxillofac Surg.

[19] Nitzan DW (2006). Arthrocentesis–incentives for using this minimally invasive approach for temporomandibular disorders. Oral Maxillofac Surg Clin North Am.

[20] Vos LM, Stegenga B, Stant AD, Quik EH, Huddleston Slater JJ (2018). Cost effectiveness of arthrocentesis compared to conservative therapy for arthralgia of the temporomandibular joint: a randomized controlled trial. J Oral Facial Pain Headache.

[21] Vos LM, Huddleston Slater JJ, Stegenga B (2014). Arthrocentesis as initial treatment for temporomandibular joint arthropathy: a randomized controlled trial. J Craniomaxillofac Surg.

[22] Al-Moraissi EA, Wolford LM, Ellis E, Neff A (2020). The hierarchy of different treatments for arthrogenous temporomandibular disorders: A network meta-analysis of randomized clinical trials. J Craniomaxillofac Surg.

[23] Hosgor H, Bas B, Celenk C (2017). A comparison of the outcomes of four minimally invasive treatment methods for anterior disc displacement of the temporomandibular joint. Int J Oral Maxillofac Surg.

[24] Ghanem WA (2011). Arthrocentesis and stabilizing splint are the treatment of choice for acute intermittent closed lock in patients with bruxism. J Craniomaxillofac Surg.

[25] Tang YH, Vos LM, Tuin AJ, Huddleston Slater JJR, Gareb B, van Bakelen NB, Spijkervet FKL (2023). Arthrocentesis versus non-surgical intervention as initial treatment for temporomandibular joint arthralgia: a randomized controlled trial with long-term follow-up. Int J Oral Maxillofac Surg.

